# Supplementary material for: Association between oral microbiome and seven types of cancers in East Asian population: a two-sample Mendelian randomization analysis
Source: Front Mol Biosci. 2023 Nov 21;10:1327893. doi: 10.3389/fmolb.2023.1327893 (PMC10702768; doi:10.3389/fmolb.2023.1327893)
Supplement: Supplementary file 1 [file DataSheet1.ZIP › Supplementary Materials/MR plots of saliva/saliva═╝/Gastric cancer/pheno.1414_to_gastric cancer_leave_one_out.pdf]

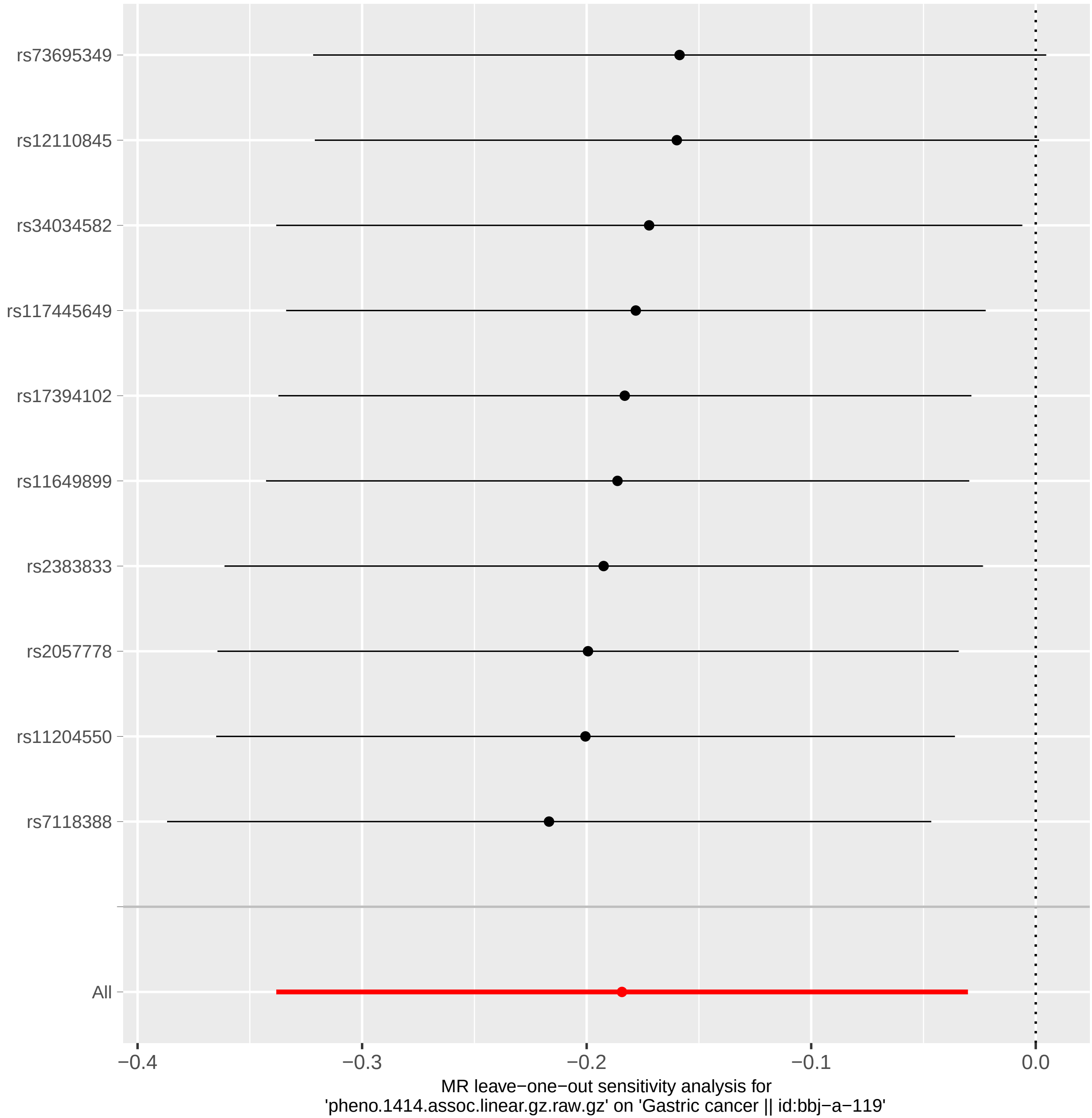
rs73695349
rs12110845
rs34034582
rs117445649
rs17394102
rs11649899
rs2383833
rs2057778
rs11204550
rs7118388
All
−0.4
−0.3
−0.2
−0.1
0.0
MR leave−one−out sensitivity analysis for
'pheno.1414.assoc.linear.gz.raw.gz' on 'Gastric cancer || id:bbj−a−119'
